# Combined topical and intravenous administration of tranexamic acid further reduces postoperative blood loss in adolescent idiopathic scoliosis patients undergoing spinal fusion surgery: a randomized controlled trial

**DOI:** 10.1186/s12891-021-04562-5

**Published:** 2021-08-09

**Authors:** Yulei Dong, Jinqian Liang, Bingdu Tong, Jianxiong Shen, Hong Zhao, Qiyi Li

**Affiliations:** Department of Orthopedic Surgery, Peking Union Medical College Hospital, Chinese Academy of Medical Science and Peking Union Medical College, Beijing, 100730 China

**Keywords:** Tranexamic acid, Spinal fusion, Adolescent idiopathic scoliosis, Blood transfusion

## Abstract

**Background:**

To indicate whether combined topical and intravenous (IV) administration of tranexamic acid (TXA) could further reduce the blood loss after surgery for adolescent idiopathic scoliosis (AIS) compared with IV-TXA alone.

**Methods:**

Ninety AIS patients who underwent posterior spinal fusion were prospectively randomized to combined group (IV + topical- TXA group) and IV-TXA alone group. TXA was infused at a loading dose of 1 g from the beginning of the surgery with a maintenance dose of 10 mg/kg/h until the wound was closed. In the combined group, 2 g TXA was injected retrogradely through a drain, while an equivalent amount of normal saline was injected in the IV-TXA alone group. The drain tube was clamped for 2 h in both groups. The amount of wound drainage and transfusion rates were analyzed.

**Results:**

The drainage volume and duration of drain were significantly lower in the combined group compared with that in the IV-TXA alone group (372.0 ± 129.7 mL vs. 545.2 ± 207.7 mL, *P* < 0.001;64.7 ± 13.9 h vs. 82.0 ± 12.5 h, *P* < 0.001). Postoperative length of hospital stay was also significantly shorter in the combined group (6.5 ± 1.51 days vs. 7.95 ± 1.44 days, *P* < 0.05). Transfusion and complication rates were comparable between the two groups .

**Conclusions:**

IV injection of TXA combined with retrograde injection of TXA into a drain and clamping it for 2 h could further reduce the total volume of drainage in AIS patients who underwent spinal fusion surgery.

**Trial registration:**

Chinese Clinical Trial Registry: ChiCTR1900024177, Registered 29 June 2019, http://www.chictr.org.cn/showproj.aspx?proj=40214

## Background

Spinal fusion is one of the major orthopedic surgeries to treat scoliosis. Substantial muscle exposure and bone resection may often cause a large volume of blood loss. Moreover, decortication of bone surface further increases the blood loss and the postoperative volume of drainage [1].As the risk of allogeneic blood transfusion (ABT)-transmitted viruses was reduced to quite low levels in the United States, transfusion-related acute lung injury (TRALI), hemolytic transfusion reactions (HTRs), and transfusion-associated sepsis (TAS) emerged as the leading causes of ABT-related deaths [2]. Moreover, ABT contributes to longer hospital stay and increased costs of hospitalization [3]. Tranexamic acid (TXA) is a synthetic lysine analogue. It takes antifibrinolytic effect by blocking the lysine binding sites on plasminogen molecules. TXA has been proved to be effective in reducing blood loss in various orthopedic surgeries, and has also been recommended in multiple guidelines for pediatric patients with high risk of blood loss [4–8]. However, after systemic application of TXA, only a small percentage of intravenously injected TXA could reach the target location. Topical use of TXA could directly target the bleeding sites and avoid the potential systemic adverse effects [9]. In a previous research, the author assessed local application of TXA by injecting it through drain tube. They concluded that topical application of TXA via a wound drain tube and clamping it for 1 h could effectively decrease the postoperative blood loss and the hospital stay in degenerative scoliosis surgeries [10]. We, in the present study, aimed to indicate whether combined intravenous (IV) and topical injection of TXA via a drain tube and clamping it for 2 h could further reduce the postoperative blood loss in adolescent idiopathic scoliosis (AIS) cases who underwent spinal fusion surgery.

## Materials and methods

### Study design

This prospective parallel randomized controlled trial (RCT,1:1) was registered at 29/06/2019 on Chinese Clinical Trial Registry (No.ChiCTR1900024177). The study was conducted between July 2019 and August 2020 in one of the largest spinal deformity centers in China. The study protocol was approved by the Institutional Review Board of Peking Union Medical College Hospital (ZS-1889) and all methods were performed in accordance with the relevant guidelines and regulations. Informed consent was obtained from parent and/or legal guardian for subjects are under 16. Before the study, a power analysis was carried out. A 20% difference in postoperative blood loss was considered as the end point for statistical analysis. With consideration of a (false-positive) and b (false-negative) levels equal to 0.05, 39 patients at least should be involved in each group; thus, 45 patients were assigned to each group considering the potential lost follow-up. A total of 90 patients were enrolled in this study. Patients were randomized to combined IV and topical administration of TXA group (IV + topical-TXA group) and IV-TXA alone group, in the operating room using sequentially numbered, opaque, sealed envelopes. Prior to data collection, a research assistant utilized a computer-generated random number table to label envelopes, with the same unique subject identification number to ensure confidentiality for all subjects. Researchers who enrolled patients and analyzed data did not take part in patients’ clinical assessment.

#### Inclusion and exclusion criteria

Patients who were diagnosed as AIS and underwent posterior spinal fusion with segmental instrumentation were included in this study. Exclusion criteria were as follows: (1) patients who underwent non-fusion surgeries (e.g., growing rod surgery), (2) patients with history of undergoing spinal fusion surgery, (3) patients with anemia or coagulation abnormalities, (4) patients with allergy to TXA.

#### Surgical procedures and interventions

We adopted controlled hypotension strategy during the surgeries. The mean arterial pressure (MAP) was controlled at 20% below the preoperative value. Cell saver was used routinely for all the cases. The surgeries were performed via a middle posterior approach by the same spine surgeon in our hospital. After exposure of the lamina and facet joints, pedicle screws were inserted by free-hand. Osteotomy was only performed on rigid cases. After the correction completed, bone chips obtained from resected spinous process and allogeneic cancellous bone chips were used as bone graft. A wound drain tube was placed under the fascia and the suture was done by layers. TXA was infused at a loading dose of 1 g from the beginning of the surgery with a maintenance dose of 10 mg/kg/h until the wound was closed. In the IV + topical-TXA group, once the suture of the skin completed in the operation room, 2 g TXA was injected retrogradely through a drain. In the IV-TXA alone group, an equivalent amount of normal saline was injected into the drain tube. The drain tube was clamped for 2 h in both groups and removed when the daily drainage volume was less than 20 mL/8 h.

Intraoperative blood loss included the amount of blood in the suction container and soaked sponge. Postoperative blood loss was assessed according to the drainage volume. Arterial blood gas analysis was performed by the anesthesiologists in the surgery room if the estimated blood loss was over 500 ml or more. Blood test was carried out at each morning 3 days after surgery and before being discharged from hospital. The trigger of ABT was set as hemoglobin level lower than 70 g/L. When the hemoglobin level was 70–100 g/L, ABT would be performed if patient had a symptom of anemia,such as lower blood pressure and increased heart rate after enough fluids had been infused. Fresh frozen plasma, platelets, cryoprecipitate, or albumin were not included in ABT. Erythropoietin (EPO, 10,000 U/day) and intravenous iron sucrose (100–200 mg/day) were given routinely after operation before discharged.

#### Outcome measures

Patients’ demographic and clinical data (e.g., blood transfusion rate, the postoperative volume of drainage, levels of hemoglobin, coagulation function, and postoperative complications) were collected and analyzed. The primary endpoint was postoperative blood loss. The secondary endpoints were transfusion rates.

### Statistical analysis

Statistical analysis was performed using SPSS 16.0 software (IBM, Armonk, NY, USA). All the continuous variables were expressed as mean ± standard deviation (SD). Independent t-test was employed to compare differences between the IV + topical-TXA group and the IV-TXA alone group. Categorical variables were analyzed by using the Pearson’s chi-squared test or Fisher’s exact test. Statistical significance was considered when *P* < 0.05.

## Results

**Fig. 1:**
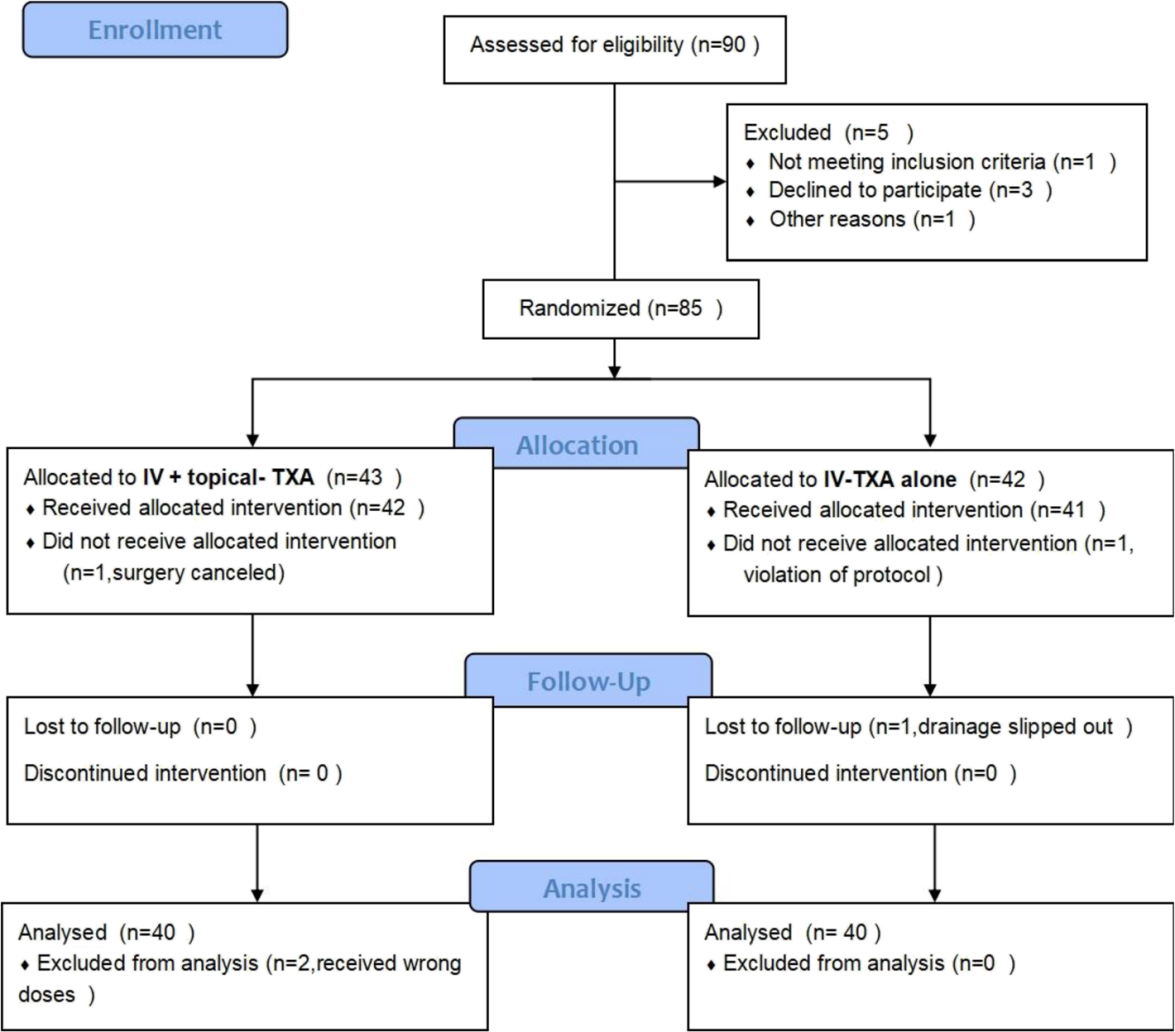
CONSORT 2010 Flow Diagram

The study was conducted between July 2019 and August 2020.A total of ninety patients were screened and 5 patients were excluded. Of the five excluded cases, syringomyelia was found in one patient, 3 patients declined to participate in the study and one patient had suspected allergy history to TXA. During the allocation step, one patient in the experimental group was excluded because the surgery was canceled as a result of fever. In the control group, one patient was excluded because the violation of protocol. During the follow up period, the drain tube of one patient in the control group slipped out. Two patients in the experimental group received wrong doses of TXA and were excluded from the final analysis. A total of eighty patients were included in the final analysis. The flowchart of patients’ selection is shown in Fig. 1. The patients’ age, body mass index (BMI), gender, preoperative hemoglobin levels, coagulation profile and platelet count were compared between the two groups (Table 1). Lenke classification, spinal fusion segments, Cobb angle of the major curve, number of osteotomies and duration of the surgeries were also compared between the two groups. There were no significant differences between the two groups.

**Table 1:** Demographic and surgical parameters between the two groups

	IV + topical-TXA group(*n* = 40)	IV-TXA alone group(*n* = 40)	*P* value
Age (year)	14.4 ± 1.9	14.0 ± 2.0	0.308
BMI (kg/m2))	19.7 ± 2.9	20.8 ± 2.6	0.062
Gender, male/total (%)	11/40	15/40	0.340
Preoperative HGB(g/L)	129.4 ± 15.2	134.8 ± 9.6	0.061
Preoperative PT	10.6 ± 1.5	11.2 ± 1.8	0.367
Preoperative APTT	25.6 ± 3.2	27.1 ± 2.7	0.558
Preoperative D-dimer	0.48 ± 0.1	0.33 ± 0.1	0.226
Lenke classification			0.785
1	15	17	
2	8	5	
3	5	4	
4	2	1	
5	10	12	
6	0	1	
Preoperative blood platelet number (10^9^/L)	193 ± 97	221 ± 88	0.128
Fusion segments	10.0 ± 2.3	10.2 ± 3.1	0.681
Cobb angle of main curve	55.3 ± 6.0	53.1 ± 7.1	0.139
Proportion of osteotomy(%)	15/40	13/40	0.639
Average number of osteotomy sites	2.0 ± 0.3	1.9 ± 0.2	0.074
Duration of the surgery (min)	186 ± 25	198 ± 37	0.774

The values of postoperative parameters are summarized in Table 2. The wound drainage at each 24 h or total drainage volume before removal was significantly lower in the IV + topical-TXA group compared with that in the IV-TXA alone group (372.0 ± 129.7 mL vs. 545.2 ± 207.7 mL, *P* < 0.01). Duration of drain was significantly shorter in the IV + topical-TXA group compared with that in the IV-TXA alone group (64.7 ± 13.9 h vs. 82.0 ± 12.5 h, *P* < 0.01). However, the transfusion rate was comparable between the two groups (4/40 vs. 5/40, *P* = 0.723). Intraoperative blood loss and cell salvage were comparable between the two groups as well. Postoperative length of hospital stay was significantly shorter in the IV + topical-TXA group than that in the IV-TXA alone group (6.5 ± 1.51 days vs. 7.95 ± 1.44 days, *P* < 0.05). The complication rate was comparable between the two groups. The complications included superficial wound infection, urinary infection, malposition of the pedicle screws. No symptomatic deep vein thrombosis was observed in none of the patients.

**Table 2:** The comparison of postoperative parameters between the two groups

	IV + topical-TXA group(*n* = 40)	IV-TXA alone group(*n* = 40)	*P* value
wound drainage at first 24 h (ml)	192.0 ± 89.7	265.2 ± 102.3	< 0.01
wound drainage at second 24 h (ml)	103.0 ± 79.7	155.6 ± 97.9	< 0.05
wound drainage at third 24 h (ml)	62.5 ± 85.4	105.3 ± 84.5	< 0.05
Total wound drainage before removal (ml)	372.0 ± 129.7	545.2 ± 207.7	< 0.01
Duration before drainage removal(h)	64.7 ± 13.9	82.0 ± 12.5	< 0.01
Intraoperative Blood loss (ml)	444.4 ± 119.3	460.3 ± 79.6	0.486
Transfusion rate(%)	4/40	5/40	0.723
Intraoperative cell salvage, ml	244.0 ± 70.8	270.2 ± 60.7	0.079
Postoperative length of stay (days)	6.50 ± 1.51	7.95 ± 1.44	0.038
Complications rates(%)	4/40	5/40	1.000

The results of postoperative blood test are summarized in Table 3. The hemoglobin level was equivalent between the two groups. Prothrombin time (PT) at postoperative day 3 (POD3) was longer in the IV + topical-TXA group than that in the IV-TXA alone group (12.7 ± 0.6 vs. 12.4 ± 0.8, *P* = 0.030). Activated partial thromboplastin time (APTT) at POD3 was shorter in the IV + topical-TXA group than that in the IV-TXA alone group (29.5 ± 1.6 vs. 31.2 ± 3.9, *P* = 0.015). APTT at POD5 was shorter in the IV + topical-TXA group compared with that in the IV-TXA alone group (27.2 ± 1.6 vs. 29.5 ± 2.6, *P* = 0.000). D-dimer levels at POD1 and POD3 were lower in the IV+ topical-TXA group than those in the IV-TXA alone group, however, they became comparable at POD5.

**Table 3:** The results of postoperative blood test between the two groups

	IV + topical-TXA group(*n* = 40)	IV-TXA alone group(*n* = 40)	*P*
HGB POD1, g/L	111.9 ± 11.6	111.1 ± 9.6	0.738
HGB POD2, g/L	109.3 ± 10.8	108.1 ± 11.5	0.297
HGB POD3, g/L	106.1 ± 11.2	104.1 ± 10.1	0.410
HGB POD5, g/L	106.9 ± 10.7	104.0 ± 10.4	0.255
PT POD1,	12.3 ± 0.6	12.3 ± 0.7	0.879
PT POD3,	12.7 ± 0.6	12.4 ± 0.8	0.030
PT POD5,	12.2 ± 0.6	12.4 ± 0.8	0.311
APTT POD1,	27.8 ± 2.3	27.9 ± 2.0	0.951
APTT POD3,	29.5 ± 1.6	31.2 ± 3.9	0.015
APTT POD5,	27.2 ± 1.6	29.5 ± 2.6	< 0.01
D-dimmer POD1	1.5 ± 0.9	2.1 ± 1.6	0.025
D-dimmer POD3	1.5 ± 0.7	2.2 ± 1.2	< 0.01
D-dimmer POD5	2.4 ± 1.0	2.6 ± 1.2	0.552

## Discussion

Scoliosis correction surgeries cause lots of bleeding. There are several blood saving techniques in surgery for scoliosis. These include iron sucrose and erythropoietin use, preoperative autologous blood donation, Intraoperative controlled hypotension, cell salvage and retransfusion, antifibrinolytic administration, and appropriate surgical techniques. Intravenous application of antifibrinolytic drugs could reduce blood loss in patients who underwent surgeries [4]. In cardiac and arthroplasty surgeries, topical use of TXA has been proved as effective as intravenous TXA, especially for the prevention of postoperative bleeding [11]. For the surgery of scoliosis, the bone resection and spinal fusion could cause a large volume of postoperative drainage. Topical use of TXA via a drain has several advantages. Firstly, the local concentration of TXA could be quite high and the drug could directly target the bone and soft tissue surface. Secondly, after local injection, a drain was clamped for 2 h, which allowed a long-term efficacy compared with local anesthetic infiltration during surgery.

Several studies have concentrated on topical administration of TXA in spinal fusion. Sudprasert et al. assessed the effects of topically applied TXA on postoperative blood loss of neurologically intact patients with thoracolumbar spine trauma. In that research, a solution with 1 g of TXA (20 mL) was administrated to the surgery site via a drain tube after the fascia was closed and the drain was clamped for 2 h later. Their results showed that the rate of postoperative packed red cells (PRC) transfusion was significantly lower in topical TXA group than that in control group, and mean total drainage volume was markedly lower in topical TXA group than that in control group [12]. Ren et al. explored the effects of topical use of TXA (tTXA) on hidden blood loss (HBL) during primary posterior lumbar spinal fusion surgery. According to their findings, in tTXA group, wound surface was soaked with TXA for 5 min before wound closure. It was also found that total blood loss, postoperative blood loss, and HBL were remarkably lower than those in control group [13]. In our previous study on topical administration of TXA in degenerative lumbar scoliosis surgery, dosage of topical TXA was 1 g and a drain was clamped for 1 h [7]. We increased the TXA dosage and duration of clamping because of a relatively longer incision in surgeries for AIS. In the present study, the efficacy of combined IV and topical administration of TXA and IV-TXA alone was compared. Because of the short half-life of TXA, IV-TXA alone was not effective postoperatively. Especially after the instrumentation, a decortication of the lamina was prepared for bone grafting, which might cause a significantly higher amount of postoperative blood loss. Retro-injection of TXA was conducted via a drain and clamping it for 2 h would allow a high concentration of local TXA and decrease the volume of drainage. Zhang et al. carried out a meta-analysis of RCTs to compare the efficacy and safety of combined IV and topical injection of TXA with IV-TXA alone for controlling blood loss in patients following primary total hip arthroplasty. They pointed out that combined topical and IV injection of TXA was a relatively effective hemostasis method compared with IV-TXA alone [14]. Pong et al. carried out a retrospective study on the effects of TXA on patients who had undergone surgical correction of adult spinal deformity. According to their findings, monitoring of the levels of D-dimer and fibrinogen during spinal surgery indicated that TXA impedes the fibrinolytic pathway by decreasing consumption of fibrinogen and clot dissolution as evidenced by the reduced formation of D-dimer [15]. In the present study, the D-dimer level was significantly lower in the IV + topical-TXA group at POD1 and POD3. This could be attributed to the effects of TXA on decreasing the levels of fibrinogen and clot dissolution.

In the present study, the drainage volume was significantly lower in the IV + topical-TXA group compared with that in the IV-TXA alone group (372.0 ± 129.7 mL vs. 545.2 ± 207.7 mL, *P* < 0.01). However, the postoperative level of HGB show no statistical significance between the two groups. This may be attributed to the low hematocrit (HCT) levels of the wound drainage whose difference could not reach statistical significance. Although the postoperative HGB level were comparable, lower would drainage could increase the recovery and rehabilitation rate and decrease the hospital stay, which was beneficial to the patients.

The current study contains a number of limitations. Firstly, the TXA plasma concentration was not detected; however, according to the literature [16], the dosage was safe, and we did not observe any adverse effects and symptomatic thromboembolic events. Secondly, hematocrit (HCT) levels of the wound drainage were not measured. Thirdly, screening tests for deep vein thrombosis (e.g., ultrasonography) were not employed. Thus, a deep vein thrombosis might exist without any obvious symptoms.

## Conclusions

IV injection of TXA combined with retrograde injection of TXA into a drain and clamping it for 2 h could further reduce the total amount of drainage in AIS patients who underwent spinal fusion surgery.

## Data Availability

The datasets used and/or analysed during the current study are available from the corresponding author on reasonable request.

## Abbreviations

ABT: Allogeneic blood transfusion
AIS: Adolescent idiopathic scoliosis
APTT: Activated partial thromboplastin time
BMI: Body mass index
EPO: Erythropoietin
HBL: Hidden blood loss
HCT: Hematocrit
HTRs: Hemolytic transfusion reactions
IV: Intravenous
MAP: Mean arterial pressure
POD: Postoperative day
PRC: Packed red cells
PT: Prothrombin time
RCT: Randomized controlled trial
TAS: Transfusion-associated sepsis
TRALI: Transfusion-related acute lung injury
TXA: Tranexamic acid
